# Morphologic, dynamic and high-resolution microscopy MRI in early-onset spondyloarthritis finger dactylitis

**DOI:** 10.1007/s00256-022-04218-y

**Published:** 2022-11-04

**Authors:** Enrico Scarano, Michele Gilio, Gianfranco Belmonte, Francesco Borraccia, Angela Padula, Giuseppe Guglielmi, Salvatore D’Angelo

**Affiliations:** 1 Radiology Department, San Carlo Hospital of Potenza, Potenza, Italy; 2 Rheumatologist Infectious Diseases Unit-San Carlo Hospital of Potenza, Potenza, Italy; 3 Rheumatology Institute of Lucania (IRel), The Rheumatology Department of Lucania, San Carlo Hospital of Potenza and Madonna Delle Grazie Hospital of Matera, Potenza and Matera, Italy; 4 Department of Clinical and Experimental Medicine, Foggia University School of Medicine, Viale L. Pinto 1, 71121 Foggia, Italy; 5 Radiology Unit, ‘‘Dimiccoli’’ Hospital, Viale Ippocrate 15, 70051 Barletta, Italy; 6 Radiology Unit, Hospital ‘‘Casa Sollievo Della Sofferenza’’, San Giovanni Rotondo, Viale Cappuccini 2, 71013 Foggia, Italy

**Keywords:** Dactylitis, Microscopy MRI, PsA psoriatic arthritis, SpA spondyloarthritis, Sausage-like digit

## Abstract

**Objective:**

Up to now, the pathophysiology of SpA dactylitis has not been entirely clarified. It is not clear which are the involved tissues and which is the primary lesion of the “sausage-like” digit. The aim of our study was to examine the finger structures in early-onset finger dactylitis using high-resolution microscopy MRI together with morphologic and dynamic MRI.

**Subjects and methods:**

In a 6-month period, 13 SpA patients (7 females and 6 males), mean age 54.07 years (range 37–73 years) and mean disease duration 7.07 years (range 1–44 years) with early-onset finger dactylitis (less than 3 months) were recruited. Nine patients had PsA, 3 HLA-B27-positive uSpA and 1 HLA-B27-negative uSpA. One patient had 2 dactylitis fingers. Ten healthy volunteers matched for age and sex with no personal and family history of SpA were enrolled. All dactylitis fingers and randomly selected fingers of the normal control subjects were imaged by morphologic, dynamic and high-resolution microscopy MRI.

**Results:**

We have found flexor tenosynovitis in all the 14 dactylitis fingers, joint synovitis in 5 and oedema in the finger soft tissue in 10. In 2 dactylitis fingers, there was oedema at the insertion of the joint capsule suggesting enthesitis. In 5 dactylitis fingers, there was only mild enhancement at the enthesis organ (collateral ligament, flexor and extensor tendons).

**Conclusions:**

Our MRI study on early-onset dactylitis demonstrates that flexor tenosynovitis, joint synovitis and oedema of the digit soft tissue are the predominant alterations visible in the early phase of evolution of dactylitis and that, therefore, enthesitis may not be considered the primary lesion of dactylitis.

## Introduction

Spondyloarthritis (SpA) dactylitis, also known as “sausage-shaped” digit, is a specific manifestation of SpA, a group of interrelated diseases which includes in addition to primary ankylosing spondylitis (AS), psoriatic arthritis (PsA), reactive arthritis (ReA), arthritis associated with inflammatory bowel disease (IBD) and the undifferentiated forms (uSpA) (1,2). Dactylitis was included, due to its high specificity and satisfactory sensitivity, among the criteria suggested by the Assessment of SpondyloArthritis International Society (ASAS) for axial (3) and peripheral SpA (4) as well as in the ClASsification criteria for Psoriatic ARthritis (CASPAR) (5).

Dactylitis is the inflammation of a finger or toe. This term is used only in some well-defined entities. These differ in the involved tissue of the digit and in the type of involvement (only the bone, only the soft tissues or both of these). They may be classified according to aetiopathogenesis as non-inflammatory (sickle cell dactylitis), inflammatory infectious (tuberculous dactylitis, syphilitic dactylitis and blistering distal dactylitis) and inflammatory non-infectious (sarcoid dactylitis and spondyloarthritis dactylitis) (1).

Up to now, the pathophysiology of SpA dactylitis has not been entirely clarified (6). It is not clear which are the involved tissues and which is the primary lesion of the “sausage-like” digit (6,7). Since obtaining histologic samples of the digital structures is not feasible, ultrasound (US) and magnetic resonance imaging (MRI) have been used to examine the multiple tissue compartments.

The first US and MRI studies established that dactylitis is due to flexor tenosynovitis and marked adjacent soft tissue swelling with a variable degree of joint synovitis (8–11).

Subsequently, a controversy arose on the role of enthesitis in dactylitis (12–16). Some MRI studies found the involvement of the entheses (12–14), while others found no evidence of the involvement of such structures (15–16).

Recently, Tan et al. used high-resolution MRI to visualize the small entheses of the digit in dactylitis and presented proof of concept for a connection between dactylitis and “digital polyenthesitis” (14).

The aim of our study was to examine the finger structures in early-onset finger dactylitis using high-resolution microscopy MRI together with morphologic and dynamic MRI.

## Subjects and methods

During a 6-month recruitment period, all consecutive patients who had finger dactylitis lasting less than 3 months (early dactylitis) and met the ASAS criteria for axial (3) and peripheral SpA (4) and/or the CASPAR criteria for PsA (5) seen at the Rheumatology Institute of Lucania (IReL) and at the Rheumatology Department of Lucania were considered suitable candidate for the study. “Sausage-shaped” fingers (dactylitis) were taken into consideration if there were the aspects suggested by Rothschild et al.: “uniform swelling such that the soft tissues between the metacarpophalangeal and proximal interphalangeal, proximal and distal interphalangeal joints, and/or distal interphalangeal joint and digital tuft are diffusely swollen to the extent that the actual joint swelling could no longer be independently recognized” (17). Exclusion criteria were systemic steroids administration, steroid injections in the flexor synovial sheaths and hands trauma in the previous 4 weeks.

During the study period, 13 patients (7 females and 6 males), age range 37–73 years (mean 54.1 years) and disease duration range 1–44 years (mean 7.1 years) were recruited. Nine patients had PsA, 3 HLA-B27-positive uSpA and 1 HLA-B27-negative uSpA. One patient had 2 dactylitis fingers. Ten healthy volunteers matched for age and sex with no personal and family history of SpA were enrolled. In such group we studied the 3rd finger because it was the digit most frequently involved in the recruited patients.

The study was approved by the local ethics committee, and all subjects gave their informed consent.

### Magnetic resonance imaging

The study was conducted on a 1.5 T Achieva scanner (Philips Medical Systems, Best, the Netherlands). All dactylitis fingers and randomly selected fingers of the normal control subjects were imaged by morphologic, dynamic and high-resolution microscopy MRI.

Patients were placed in prone position with the arm extended over the head. The hand and the wrist were tightly fixed with sandbags. A one-channel receive/transmit hand-wrist-coil was applied. After placement of gradient-echo localizers, the following sequences were used: coronal T1-weighted 2D spin-echo images, obtained for the assessment of the anatomy of most of the musculoskeletal structures (joints, tendons, collateral ligaments, etc.), coronal and sagittal STIR T2-weighted 2D fast spin-echo images of the fingers involved [TSE TR 2225 ms, TE 80 ms, TI 145 ms, FOV 16–18 cm, thickness 3 mm, NEX 3, matrix 256 × 224 (rec matrix 352), voxel dimension 0.625 (Rec vox 0.57)] and axial DP SPIR of the hand [TSE TR 4234 ms, TE 25 ms, FOV 14 cm, thickness 3 mm, matrix 232 × 224, voxel dimension 0.625 (Rec vox 0.44)], obtained to identify areas of oedema, as well as fluid in the joint space and in tenosynovial sheaths.

Subsequently, a 3D-encoded spoiled gradient echo sequence (THRIVE T1) with fat saturation [3DFFE T1 TR/TE 7/3.4 ms, matrix 100 × 152, NEX 2, voxel dimension 1 × 1 × 2 mm Rec vox 0.9 × 0.9 × 1 mm] was used for measurement of the time course of contrast-medium uptake in the tissues. After bolus injection of 0.2 mmol of gadoterate meglumine (Dotarem, Guerbet) per kilogram of body weight, with an injection rate of 2 mL/s, 15 acquisitions were performed within 4 min and 30 s. Images obtained were used to demonstrate areas of permeability in the soft tissue and bone. Seven minutes after contrast-medium injection, another T1-weighted fat-suppressed axial 3D THRIVE acquisition was performed for late enhancement.

Subsequently, a 47-mm diameter microscopy MRI surface coil was applied for the imaging of the affected fingers to cover at least the tissues between the proximal and distal interphalangeal joints and the insertion of the flexor and extensor tendons. Three-dimensional gradient-echo sagittal T1 high-resolution images [3DFFE T1 TR/TE 42/11 ms, FOV 6 × 4.8 cm, NEX 2, voxel dimension 0.3 × 0.3 × 1.5 mm, Rec vox 0.12 × 0.12 × 0.75 mm] were acquired using the microscopy MRI surface coil to best-bit areas of inflammation in the soft tissue and bone. The images were reconstructed along the coronal and axial plane to evaluate the insertions of the capsule and collateral ligaments.

The total examination time for the patients as well as the normal controls was 45 min.

### Image analysis

The following anatomic structures were evaluated: capsule/synovium, extracapsular soft tissue, flexor and extensor tendons, collateral ligament insertions, bone cortex, bone marrow, finger pulleys, enthesis organ (collateral ligament, flexor and extensor tendons), finger volar plates and finger soft tissue.

Alterations were evaluated in a dichotomous manner (present/absent) by two radiologists with 20 years of experience in musculoskeletal radiology (ES and FB), who worked separately and were blinded to the clinical status of the study subject. Signal intensity changes were scored on a scale between 0 (absence) and 3 (severe). Images on which the observers were in disagreement were examined together, and a reconciled grade was established.

Bone erosions were recorded when a cortical break was evident on T1-weighted images. T2-weighted fat-suppressed spin-echo and PD images were used to identify bone marrow oedema and synovial fluid distension; the images were compared with the gadolinium-enhanced images to distinguish between bone oedema (enhancing) and encysted fluid (no enhancing). Enthesitis was defined as increased signal intensity adjacent at the insertion of ligaments, tendons or joint capsule. Regions of enthesitis, tenosynovitis, arthritis or capsulitis were defined on images acquired after the dynamic injection of the contrast agent. The presence of high signal intensity within the enthesis, the insertional bone, the tenosynovial sheaths, the synovial capsule and the soft tissue on post-contrast images was used to score inflammation of that structure. The rate of enhancement was evaluated by a radiologist on the slice that showed the highest visual enhancement. It was calculated as Δ on a small, elliptical region of interest (ROI) of approximately 6–10 mm^2^ positioned in the area of the highest visual enhancement. We have assigned a multimodal score from 0 to 3: three if it presents an enhancement of ≥ 100% compared to the basic conditions, 2 if it was between 50 and 100% and 1if it is was lower than 50%. The analysis of T1 3D high-resolution images for all structures was used to confirm or exclude the presence of late-enhancement, expression of inflammatory permeability. It was calculated by positioning a small elliptical region of interest (ROI) of approximately 6–10 mm^2^ in the sensible sites previously identified.

### Statistical analysis

Statistical analysis was not performed since no abnormalities were seen in the fingers of healthy controls.

## Results

### Morphological alterations

**Fig. 1:**
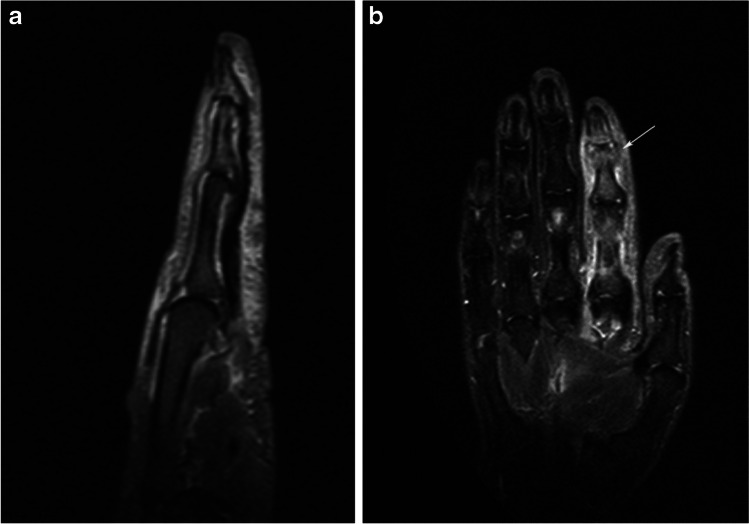
**a** and **b** Sagittal and coronal STIR T2 image of the second right finger of a 50-year-old woman showing fluid/synovitis in the flexor synovial sheaths and diffuse subcutaneous oedema. There is also distension of the capsule of distal interphalangeal joint (white arrow)

**Fig. 2:**
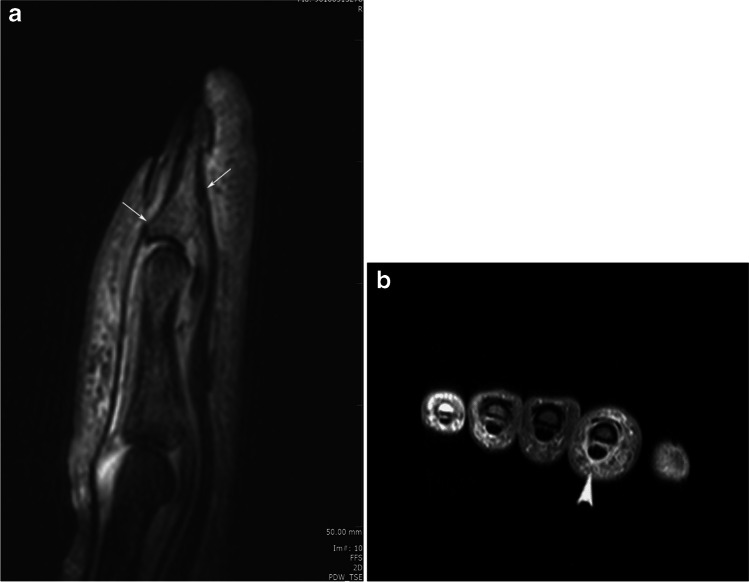
Sagittal PD fat sat (**A**) and axial PD fat sat (**B**) images of the same finger in Fig. 1 showing flexor tenosynovitis and soft tissue oedema (arrowhead). There is no evident of bone oedema at the insertion of the flexor and extensor tendons in this image (arrows). The axial image shows inflammation adjacent to the finger pulley of the 2nd finger

**Fig. 3:**
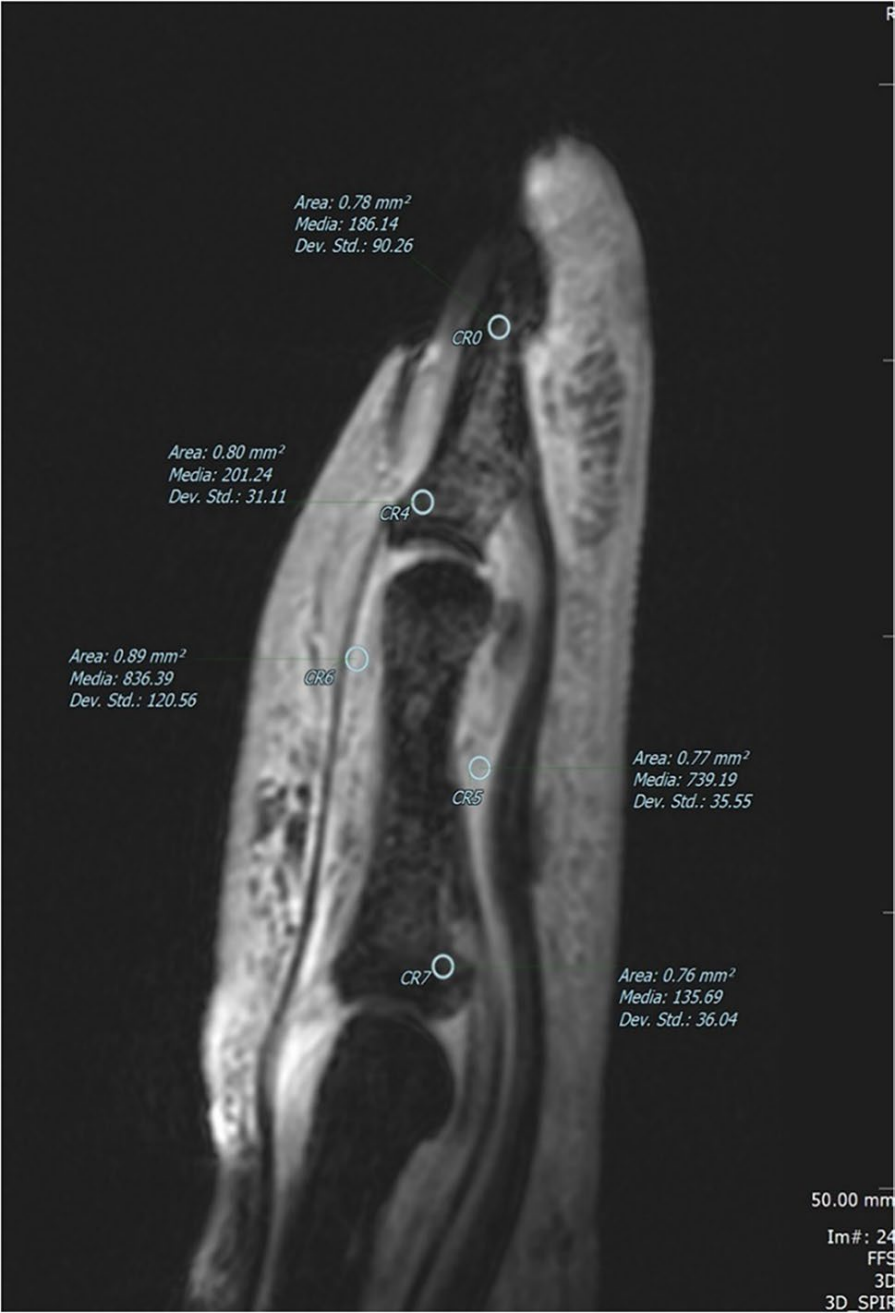
Sagittal 3D high-resolution T1-weighted with fat saturation of the same finger in Figs. 1 and 2, obtained after the dynamic phase, by using a 47-mm diameter microscopy surface coil. The ROIs (○) were positioned at the enthesitis sites, in synovial sheaths and in the normal bone showing high-intensity signal of synovial sheaths and intensity similar to that of the normal bone at the enthesis insertion

**Fig. 4:**
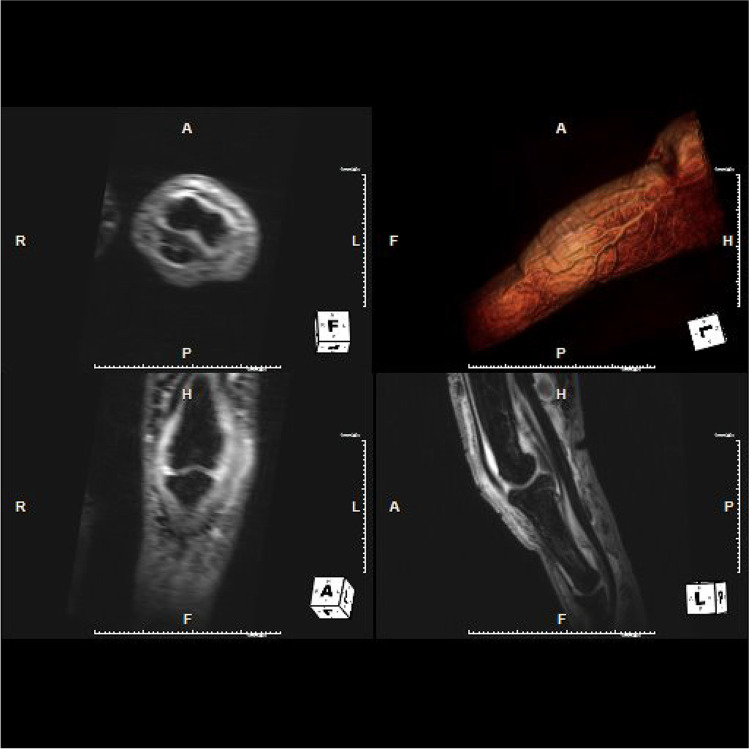
MPR reconstruction on 3 planes of 3D high-resolution T1-weighted with fat saturation of the second right finger of a 43-year-old man. There is distension and enhancement of capsule/synovium of the proximal interphalangeal joint. There is flexor and extensor tenosynovitis and enhancement of their synovial sheaths without enhancement at the enthesis

**Fig. 5:**
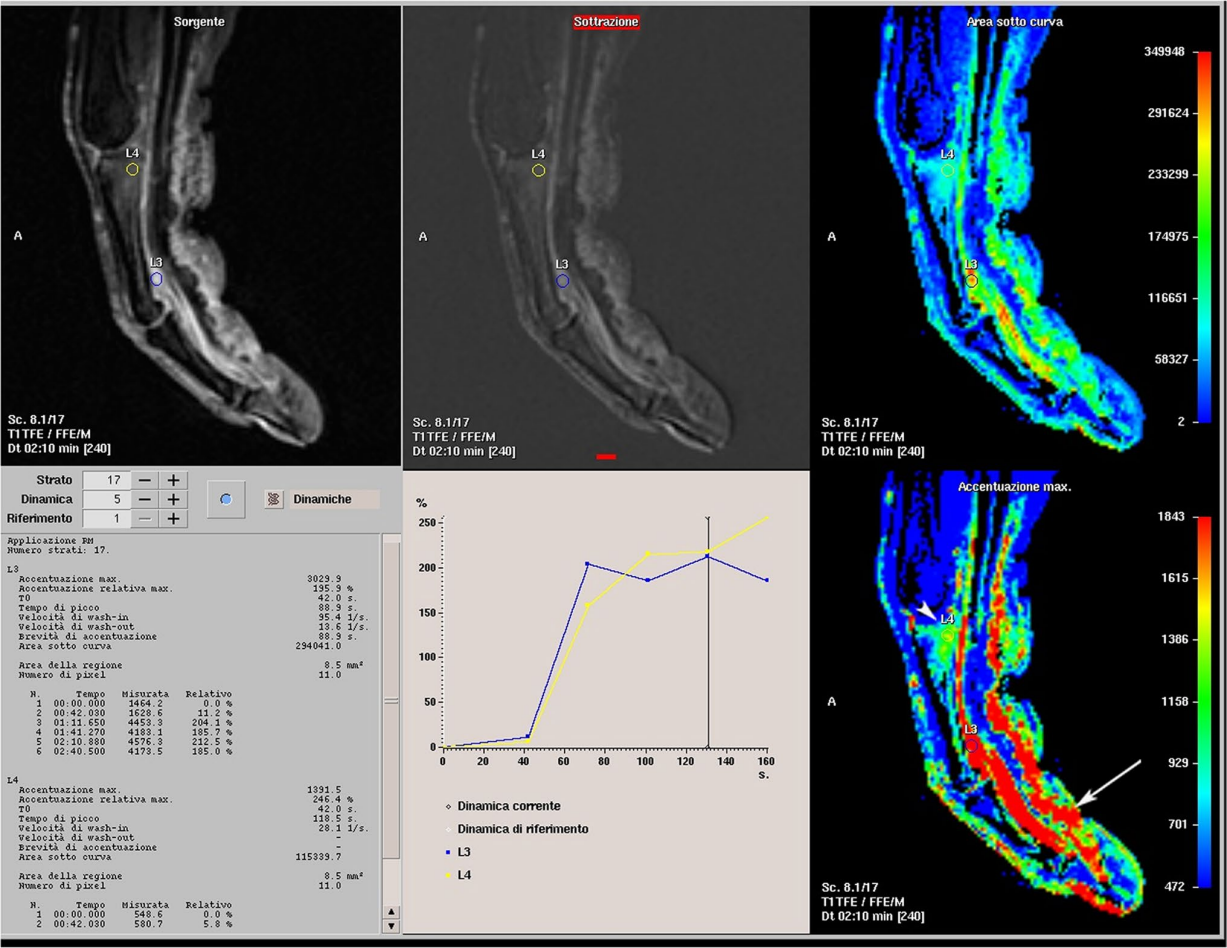
Sagittal T1-weighted sagittal image with fat saturation obtained during the course of the contrast medium uptake of a dactylitis finger of a 59-year-old man. There is enhancement of the flexor tendon synovial sheaths (arrow) and of a functional enthesis at the base of the proximal phalange (arrowhead)

In the 7 joints (five patients) out of the 14 dactylitis fingers, there was fluid distension (Table 1, Fig. 1). In two out of these joints, there were signs of enthesitis at the capsule insertions, and in other two, there were small erosions. There was some fluid in the tendon sheaths of all the dactylitis fingers (Figs. 1, 2, 3, 4 and 5). Bone oedema was seen at the pulleys in the 2 dactylitis fingers (Fig. 2B) but not at the insertions of the flexor and extensor tendons or in other sites of the enthesis organ (collateral ligament, flexor and extensor tendons). Ten out of the 14 dactylitis fingers showed oedema in the digit soft tissue. No alterations were seen in the fingers of the control subjects.

**Table 1:** Morphological alterations

	SpA patients (*n* 13)	Normal subject (*n* 10)
Fluid distension of joint capsule	7 (for 5 patients) (38%)	0
Fluid distension of tenosynovial sheaths	14 (for 13 patients, 1 patient with 2 finger involved) (100%)	0
Enthesitis of capsule	2 (15%)	0
Bone oedema at tendon insertion (flex or est), finger pulleys, enthesis organ (collateral ligament, flexor and extensor tendons)	2 (15%)	0
Soft tissue oedema	10 (77%)	0
Erosions	2 (15%)	0

### Dynamic MRI alterations

Enhancement was seen in the capsule/synovium of 7 involved joints (score 3 in 3 joints and score 2 in 4), in the flexor tendon synovial sheaths of the all 14 dactylitis fingers (Fig. 5) (score 3 in 12 synovial sheaths and score 2 in 2) and at the capsular insertion of the two joints shown by the morphologic examination (Table 2). Bone enhancement was seen in 5 dactylitis fingers at the enthesis organ (collateral ligament, flexor and extensor tendons) with a score of 2 in 1 finger and of only 1 in the other 4 (Fig. 5). Enhancement was also seen at the soft tissue of the 10 fingers identified by the morphologic examination (score 2 in 6 and 1 in 4). No alterations were seen in the fingers of the control subjects.

**Table 2:** Dynamic MRI alterations

	SpA patients (*n* 13)	Normal subject (*n* 10)
Enhancement of capsule/synovium	7 (for 5 patients) (38%) (3 score 3, 4 score 2)	0
Enhancement of tenosynovial sheaths	14 (for 13 patients, 1 patient with 2 finger involved) (100%) (12 score 3, 2 score 2)	0
Bone enhancement at capsular insertion	2 (15%) (2 score 2)	0
Bone enhancement at tendon insertion (flex or est) finger pulleys, enthesis organ (collateral ligament, flexor and extensor tendons)	5 (38%) (1 score 2, 4 score 1)	0
Soft tissue enhancement	10 (77%) (6 score 2, 4 score 1)	0

### High-resolution MRI alterations

Late enhancement was seen in all the involved structures identified by the previous morphologic and dynamic sequences except for the enthesis organ (Table 3, Figs. 3 and 4). The enhancement was seen in only 2 of the five fingers identified by dynamic MRI. The examination was normal in the fingers of healthy controls.

**Table 3:** High-resolutions MRI alterations

	SpA patients (*n* 13)	Normal subject (*n* 10)
Late enhancement of capsule/synovium	7 (for 5 patients) (38%)	0
Late enhancement of tenosynovial sheaths	14 (for 13 patients) (100%)	0
Late enhancement at capsule insertions	2 (15%)	0
Bone enhancement at tendon insertion (flex or est) finger pulleys, enthesis organ (collateral ligament, flexor and extensor tendons)	2 (15%)	0
Soft tissue late enhancement	10 (77%)	0

## Discussion

Our MRI study is the first to evaluate early-onset dactylitis. We chose to take in consideration only finger dactylitis with the aim to have more homogeneous data. We used morphologic and dynamic MRI in addition to high-resolution microscopy MRI to visualize better the involved structures. In the morphologic study, we have found flexor tenosynovitis in all the 14 dactylitis fingers, joint synovitis in 5 and oedema of the finger soft tissue in 10. In only 2 dactylitis fingers, there was oedema at the insertion of the joint capsule suggesting enthesitis. Dynamic MRI showed bone enhancement in 5 dactylitis fingers in the enthesis organ. The score was low (score 2 in one finger and 1 in the other 4). The entheseal scores were remarkably low if compared to that of the flexor synovial sheaths. The possible reasons for this discrepancy can be a volume artefact or the diffusion of synovial sheath inflammation to the adjacent structures including the surface of the bone. In favour of the second motivation, there is the involvement of the enthesis organs in those finger dactylitis with the higher score of synovial sheath inflammation. In addition, high-resolution microscopy MRI, performed in the late phases of the examination, showed bone enhancement in only 2 of the 5 dactylitis fingers. Altogether, the results of our study exclude the presence of enthesitis in the majority of dactylitis fingers and a pathogenetic role of enthesitis in early-onset finger dactylitis.

A limitation of this study is the small sample size, and therefore, larger studies are needed in order to confirm our results.

The first US and MRI studies established that dactylitis is due to flexor tenosynovitis and marked adjacent soft tissue swelling with a variable degree of joint synovitis (8–11). Flexor tenosynovitis was found to be constantly present, while joint synovitis occurred in 17–66% of the “sausage-like” digits (8–11).

In the late nineties and in the following years of the new millennium, McGonagle and his colleagues conjectured that enthesitis is the primary lesion in SpA and that synovitis of the various structures (joint, tendon and bursa) represents a secondary phenomenon as a result of the release of pro-inflammatory cytokines from the inflamed entheses (18–21). In their judgement, flexor tenosynovitis of dactylitis is attributable to enthesitis as a result of the diffusion of cytokines along the tenosynovial sheaths (21). We showed, by using the fast spin-echo (FSE)-T2-weighted sequences with fat saturation, that in SpA dactylitis, there is no evidence of enthesitis of the insertion of the flexor digitorum tendons and of the attachment of the capsule of the digit joints (15,16). However, McGonagle and his colleagues suggested that in dactylitis, enthesitis could occur at the numerous “functional entheses” that the digit flexor tendons form with retinacula or pulleys (21). The term “functional enthesis” refers to the sites where tendons and ligaments wrap around bony pulleys with a contact between hard and soft tissues but without a true anchorage as at the classical entheses themselves. These “functional entheses” are frequently associated with the presence of fibrocartilage that reduce compression and shear. They advocated that this hypothesis could be tested by using high-resolution imaging.

In 2008, Healy et al. report their MRI study on psoriatic dactylitis (13). Scans were performed before and after treatment and pre- and post-gadolinium contrast. Joint synovitis and soft tissue oedema were the most frequent alterations. The oedema extended all around the circumference of the digit and was not ever associated with flexor tenosynovitis, which was, however, a common finding. Bone oedema fluctuated from small areas closest to the insertions of the joint capsule to oedema involving the entire phalanx. This last feature seemed to confirm the hypothesis by McGonagle on a primary involvement of the entheses in dactylitis.

More recently, the Leeds group used high-resolution MRI to visualize the small entheses of the digit in dactylitis (14). They found collateral ligament enthesitis, extensor tendon enthesitis and functional enthesitis in most dactylitis fingers and in none of the normal digits of healthy volunteers. In 75% of the dactylitis digits, there was flexor tenosynovitis together with flexor tendon pulley/flexor sheath micro-enthesitis suggesting a form of functional enthesitis. They concluded that the swelling along the digits observed in dactylitis is linked to polyenthesitis.

In our study, we have found no signs of enthesitis in most dactylitis fingers evaluated. It should be emphasized that our patients had early-onset dactylitis and that no involved finger had arthritis in the previous years. As a matter of fact, we did not see any deformed or limited joint in our 13 patients with sausage-like fingers. We observed enthesitis on MRI less often when dactylitis is of less than 3-month duration compared to previous reports in more advanced disease. Since enthesitis is not the primary MRI observation, occurring in only a minority of cases, this raises doubt about the unifying concept of polyenthesitis as the underlying cause of this condition. Probably, enthesis involvement develops during the course of the disease when radiological joint damage appears. Brockbank et al. have reported that dactylitis can be destructive and that radiologic progression is more frequent than in digits without the sausage-like aspect (22). The presence of erosions in the joints of two of our patients with early-onset dactylitis seems to confirm this hypothesis.

It is possible that microtrauma, as suggested by Tan et al., can have a role in the location of enthesitis in the advances phase of dactylitis (14).

In conclusion, our MRI study on early-onset finger dactylitis demonstrates that flexor tenosynovitis, joint synovitis and oedema of the digit soft tissue are the predominant alterations visible and that enthesitis may not be considered the primary lesion of dactylitis.
